# Can an Investigation of a Single Gene be Effective in Differentiating Certain Features of the Bipolar Disorder Profile?

**DOI:** 10.2174/1745017902117010187

**Published:** 2021-12-22

**Authors:** Martina Piras, Alessandra Scano, Germano Orrù, Antonio Preti, Cinzia Marchese, Goce Kalcev

**Affiliations:** 1 Innovation Sciences and Technologies, University of Cagliari, Cagliari, Italy; 2 Department of Surgical Science, Molecular Biology Service Lab,, University of Cagliari, Cagliari, Italy; 3 Department of Neuroscience, University of Turin, Turin, Italy; 4 Department of Experimental Medicine, Sapienza University of Rome, Rome, Italy

**Keywords:** Bipolar disorder, Genes, CACNA1C, FRET probe, Evolutionary model, Mental disorder

## Abstract

Bipolar disorder (BD) is amongst the most common heritable mental disorders, but the clarification of its genetic roots has proven to be very challenging. Many single nucleotide polymorphisms (SNPs) have been identified to be associated with BD. SNPs in the CACNA1C gene have emerged as the most significantly associated with the disease. The aim of the present study is to provide a concise description of SNP 1006737 variants identified by Real Time PCR and confirm sequencing analysis with the Sanger method in order to estimate the association with BD. The molecular method was tested on 47 Sardinian subjects of whom 23 were found to not be mutated, 1 was found to be a carrier of the homozygous A allele and 23 were found to be carriers of the heterozygous G allele. Moreover, the positive results of the preliminary application suggest that the development of the screener could be extended to the other 5 genetic variables identified as associated with BD.

## Dear Editor,

Bipolar disorder (BD) is amongst the most common heritable mental disorders, but the clarification of its genetic roots has proven to be challenging [1]. The genome-wide association studies (GWAS) era has modified the perception regarding this disorder [2]. Before the epoch of molecular genetics, much of the etiologic concern of BD lies in the routes of genetic epidemiology [3]. Family studies reveal that BD appears to run in families, with a 10-15% risk of mood disorder among relatives of people with BD, but the effects of shared environment from those of mutual genes could not be detected. The genetic difficulty of BD is attributed to its complex and heterogeneous clinical presentation [4]. One of the biggest issues is that genetic studies have concentrated almost completely on individuals who can be effortlessly diagnosed by interview or are already in treatment, which clearly provides an incomplete view. Genome-wide association studies have so far been the most effective strategy for identifying genetic variants related to BD [5].

One of the candidate genes most commonly associated with bipolar disorder is the CACNA1C gene. This gene encodes the alpha-1 subunit of voltage-gated calcium channel CaV1.2 [6]. Moreover, it plays a role of great importance in calcium ion influx toward cells. It is well known that calcium channels are essential components of various calcium-dependent processes, such as programmed cell death, regulation of gene expression, the release of neurotransmitters, and muscle contraction. As an L-type voltage-gated Ca^2+^ channel, CACNA1C provides the depolarization of neurons through calcium ion influx [7]. To date, certain SNPs of this gene, such as rs1006737, are strongly associated with BD. The rs1006737 is perhaps the most frequently studied CACNA1C SNP, and it is related to intracellular calcium homeostasis and amygdala activity [8, 9]. The A allele is considered to be a risk factor for BD and various studies have shown the odds ratio for BD as ranging between 1.18-1.07. Several researchers have suggested that this variant is able to impact gene expression, and others have revealed an effective variation of CACNA1C mRNA in post-mortem brain studies [10, 11].

The genetic roots of BD underpin an evolutionary basis for the persistence of the disorder in the general population despite its impact on morbidity, mortality, and quality of life [12, 13]. Affective temperaments underlie the basis for the predisposition to affective disorders. They reflect the most frequent phenotypic expression of the genes that are crucial for BD [14, 15]. According to an evolutionary model, adaptive traits are more common among “dilutive” forms of bipolar disorder or among “clinically stable” biological relatives who carry part but not all of the genes of the mentally ill proband [16]. Furthermore, affective disorders in their subthreshold forms appear to serve key functions in emotional disclosure and survival. Depressive characteristics, among other functions, would enhance the suffering of other members of the species, interacting with the established anxious temperament, and this would favor the survival of other genetically related conspecifics. On the other hand, hyperthymic attributes would provide definitive dominance in leadership, investigation, territoriality and mating. These are just some of the potential pro-survival effects of the rich temperamental characteristics promoting bipolarity within an evolutionary composition [17].

Many single nucleotide polymorphisms (SNPs) have been identified to be associated with BD. SNPs in the CACNA1C gene have emerged as the most significantly associated with the disease. The aim of the present study is to provide a concise description of SNP 1006737 variants identified by Real Time PCR and confirm sequencing analysis with the Sanger method, to estimate the association with BD.

A total of 47 Sardinian subjects were examined using oral mucosal samples collected with oral swabs and stored in 1 ml eppendorf tubes containing 400 µl of 0.5 M EDTA and dimethyl sulfoxide. 16 subjects were BD patients with comorbid conditions, such as anxiety, psychosis and depression from the Centro di Psichiatria di Consultazione e Psicosomatica (CPCP- AOU Cagliari). Overall, 9 BD patients manifested familiarity concerning bipolar disorder, anxiety, depression and psychosis. Other 31 subjects were part of the control group. Regarding them, 27 subjects were from Sardinia, while 3 Sardinians residing in France and 1 Sardinian residing in Brazil. The Committee of the “Azienda Ospedaliero-Universitaria di Cagliari”, Cagliari, Italy, approved the study on February 27^th^, 2019 (reference number NP / 2019 / 1003).

DNA was extracted using a specific extraction kit for tissue fragments (*e.g*., DNasy Blood & Qiagen Tissue Kit). The molecular system involves the use of a Fluorescence Resonance Energy Transfer (FRET) probe [18] that was evaluated by means of bioinformatics tools, designed for forecasting the molecular behavior of DNA probes used in the research field or for laboratory analysis methods. In our study, we propose the genotyping of rs1006737 with greater specificity/selectivity using a molecular procedure based on FRET probes. Compared to traditional or recent methods, such as RFLP or DNA sequencing procedures, this approach has been found to be faster, less molecular, simpler and more accurate. Particularly, FRET is a probe in which an excited donor transfers energy to an accepting group through dipole-dipole, a process that does not involve the emission of ionizing radiation [19-22]. One of the two probes has a fluorochrome capable of transferring energy to the second probe's fluorochrome which will emit light of a particular wavelength detected by real-time PCR. In this way, the fluorescence is only detected if both probes have bound to the DNA during the PCR reaction. In FRET technologies, the classic chemistry of dyes is composed of fluorescein isothiocyanate (F1) at the 3 'label of the upstream probe, while the downstream probe (acceptor) is labeled with the Red 640 fluorophore. Nucleotide variation in the target acceptor DNA leads to melting temperature (Tm).

In the PCR apparatus, this result has been visualized by a shift in the melting peak, monitoring the F2 fluorescence during the heating of the sample [23-26].

Our work is an indicator of a good possibility of allelic diversity for this gene region by evaluating the melting temperatures. To confirm the specificity of the method, some samples were analyzed by Sanger sequencing. Regarding this step of the study, we used Real-time PCR mutation analysis with FRET probes as a method used to differentiate A and G alleles in SNP rs1006737. FRET probes are able to distinguish the presence of both G and A alleles with specificity and selectivity errors (heterozygous profile).

Overall, the molecular method was tested on 47 subjects, of whom 23 were found to not be mutated, 1 was found to be a carrier of the homozygous A allele and 23 carriers of the heterozygous G allele.

## CONCLUSION

The preliminary results seem to indirectly confirm the starting hypothesis according to which a genetic condition is being explored that can give rise to contrasting phenotypes in terms of adaptation. Additionally, the proposed technique appears innovative, interesting and practicable. The preliminary application showed that the development of the screener can also be applied to the other 5 genetic variables identified (ANK3, NCAN, ODZ4, SYNE1, and TRANK1 genes); in fact, with the analysis of a single variable, there is a tendency to identify the target object, that is the screener that identifies the spectrum of bipolar disorder.
